# Systems Medicine Design for Triple-Negative Breast Cancer and Non-Triple-Negative Breast Cancer Based on Systems Identification and Carcinogenic Mechanisms

**DOI:** 10.3390/ijms22063083

**Published:** 2021-03-17

**Authors:** Shan-Ju Yeh, Bo-Jie Hsu, Bor-Sen Chen

**Affiliations:** Laboratory of Automatic Control, Signaling Processing and Systems Biology, Department of Electrical Engineering, National Tsing Hua University, Hsinchu 30013, Taiwan; m793281@gmail.com (S.-J.Y.); ee402490@gmail.com (B.-J.H.)

**Keywords:** Triple-negative breast cancer (TNBC), non-TNBC, genome-wide genetic and epigenetic network (GWGEN), systems medicine design, multi-molecule drugs

## Abstract

Triple-negative breast cancer (TNBC) is a heterogeneous subtype of breast cancers with poor prognosis. The etiology of triple-negative breast cancer (TNBC) is involved in various biological signal cascades and multifactorial aberrations of genetic, epigenetic and microenvironment. New therapeutic for TNBC is urgently needed because surgery and chemotherapy are the only available modalities nowadays. A better understanding of the molecular mechanisms would be a great challenge because they are triggered by cascade signaling pathways, genetic and epigenetic regulations, and drug–target interactions. This would allow the design of multi-molecule drugs for the TNBC and non-TNBC. In this study, in terms of systems biology approaches, we proposed a systematic procedure for systems medicine design toward TNBC and non-TNBC. For systems biology approaches, we constructed a candidate genome-wide genetic and epigenetic network (GWGEN) by big databases mining and identified real GWGENs of TNBC and non-TNBC assisting with corresponding microarray data by system identification and model order selection methods. After that, we applied the principal network projection (PNP) approach to obtain the core signaling pathways denoted by KEGG pathway of TNBC and non-TNBC. Comparing core signaling pathways of TNBC and non-TNBC, essential carcinogenic biomarkers resulting in multiple cellular dysfunctions including cell proliferation, autophagy, immune response, apoptosis, metastasis, angiogenesis, epithelial-mesenchymal transition (EMT), and cell differentiation could be found. In order to propose potential candidate drugs for the selected biomarkers, we designed filters considering toxicity and regulation ability. With the proposed systematic procedure, we not only shed a light on the differences between carcinogenetic molecular mechanisms of TNBC and non-TNBC but also efficiently proposed candidate multi-molecule drugs including resveratrol, sirolimus, and prednisolone for TNBC and resveratrol, sirolimus, carbamazepine, and verapamil for non-TNBC.

## 1. Introduction

Breast cancer is one of the most frequently diagnosed malignancies and the leading cause of cancer death in women worldwide [1]. Among all the breast cancers, triple-negative breast cancer (TNBC), with the absence of targeted therapy, is a more heterogeneous subtype of breast cancer immunohistochemically defined by lack of the expression of estrogen receptor (*ER*), progesterone receptor (*PR*), and human epidermal growth factor receptor-2 (*HER2*) [2,3]. TNBC not only constitutes approximately 10–15% of all breast cancers, but also tends to be more common in young women than other subtypes of breast cancer [4]. Patients with TNBC have the worst prognosis and mortality risk in five years than other subtypes of breast cancer and cannot benefit from hormone- or trastuzumab-based therapy because of the loss of target receptors such as *ER*, *PR*, and *HER-2* [5]. Chemotherapy is the standard therapeutic approach for patients in both the early and advanced stages of TNBC. It is noted that polypharmacology not only provides great opportunities for drug repurposing to exploit off-target effects in a new single-target indication, but also offers exciting opportunities to slow, overcome, or even prevent inherent or adaptive drug resistance through the simultaneous blockade of multiple targets or pathways [6]. Therefore, developing systematic procedure in a computational framework to discover the potential combinations of multi-molecule drugs for new therapeutic treatment of TNBC and non-TNBC is indispensable.

TNBC has a more biologically aggressive behavior, higher recurrence rate, higher frequency of metastases and worse survival than other subtypes of breast cancers. The carcinogenic molecular mechanism of cancer is often caused by aberrations of gene, microRNA (miRNA), long non-coding RNA (lncRNA), epigenetic modification, and microenvironment factors. The miRNAs are small non-coding region in RNAs of 20–22 nucleotides, which take part in all biological pathways in multicellular organisms including mammals [7]. It is known that miRNAs participate in the progression of cancers by modulating cell development, differentiation, proliferation, and apoptosis. Extensive studies have demonstrated that miRNAs are dysregulated at all stages of breast cancer and have the potential to be prognostic and predictive biomarkers [8,9,10]. Moreover, the other type of non-coding RNAs that exceed 200 nucleotides in length is lncRNAs. They are now accepted as important regulators in cancer development and different biological processes, such as chromatin remodeling and transcriptional and posttranscriptional regulation [11]. Recent evidences show that microenvironment-mediated epigenetic modification, which is the process of heritable and reversible change in gene expression that occurs without a change in the DNA sequence, plays an essential role in cancer [12,13].

Over the past decade, many researchers have been dedicated to analyzing protein–protein interactions (PPIs) combined with functional information with different algorithms. It not only improves the accuracy of protein complex detection but also broaden our understanding of biological processes mediated through protein interactions. Based on topological features of the PPI network and Gene Ontology (GO) annotations, the likelihood of a gene involving in cancer is predicted by well-known classifiers, support vector machines (SVMs) [14]. Carried out by walking on the fingerprints similarity network randomly, a multi-level protein-protein interaction network (PPIN) reconstruction method (MLPR) was proposed [15]. The clustering based on maximal cliques (CMC) algorithm with the iterative scoring method has shown to be more robust toward random noise and to achieve better performance of protein complex prediction. By applying the shortest path algorithm in PPI networks, candidate genes which are associated with formation and development of gastric cancer could be found [16]. The surge of public next generation sequencing (NGS) data availability has facilitated the application of systems biology integrating heterogeneous data to investigate various human diseases with computational modeling approaches. Based on constructing the pathway logic symbolic systems model via genomic, transcriptional, and proteomic profiles, the subnetworks within the EgfR-MAPK pathway are identified [17]. Bayesian networks, which could describe direct molecular interaction as well as indirect influences that proceed through additional unobserved components, have demonstrated to be useful for understanding the operation of cell signaling networks [18]. Moreover, one study proposed a mathematical model of the plant clock using biological hypotheses and parameters solved by optimization methods to gain insights into the clock components within core mechanisms [19]. Systems biology approaches with modeling techniques enable us to elucidate the pathways, which are critically involved in tumor formation and progression, consequences of altered cell behavior in tissue environment and effects of molecular therapeutics [20].

On average, for traditional drug discovery, the pipelines take 12 to 16 years from inception to market and cost one to two billion dollars [21]. However, drug repurposing, which is a strategy for identifying new uses for approved or investigational drugs differing from their original objective and purpose, could be done in less than half the time at a quarter of the cost [22]. The general approaches used in drug repurposing are novel data sources, retrospective clinical analysis, pathway mapping, genetics association, molecular docking and signature matching [23]. For signature matching, the connectivity map (CMap), which was established by the Broad Institute, consists of gene expression profiles generated by the dosing of more than 1300 compounds in a number of cultivated cell lines. It has widely been applied in several human disease investigations, including colon cancer, prostate cancer, breast cancer, diet-induced obesity, and Alzheimer’s disease [24,25,26,27]. Along with the drug resistance found in oncology, the need for more effective combination therapies to overcome this issue is increasing. One study has proposed a strategy to provide a testable hypothesis for combination therapies based on gene expression signatures between chemical perturbation and disease statuses [28].

In this study, we propose a systems medicine design procedure, which uses systems biology approaches for finding essential biomarkers as drug targets and propose potential candidate drugs by designing two filters considering drug regulation ability and toxicity. For systems biology approaches, firstly, a candidate protein–protein interaction network (PPIN) and a gene regulatory network (GRN) are constructed, respectively, by big database mining. Secondly, with the help of microarray data of TNBC and non-TNBC, we apply systems identification and model selection methods to obtain real GWGENs. Thirdly, the principal network projection (PNP) approach is used to extract the core GWGENs from the real GWGENs. Based on the analyses of the core signaling pathways denoted by KEGG pathway, the carcinogenic molecular mechanisms for TNBC and non-TNBC could be found as well as essential biomarkers which could be drug targets. In order to recommend candidate drugs for the selected biomarkers, we take drug regulation ability and toxicity into account.

Nowadays, few studies propose a procedure of systems medicine design from identifying potential biomarkers as drug targets for one disease to recommending its candidate drugs by designing filters. Systems medicine design procedure, an interdisciplinary approach on interpretation of heterogeneous data, gives an alternative way for drug discovery to find new therapeutic treatment of TNBC and non-TNBC.

## 2. Results

**Figure 1:**
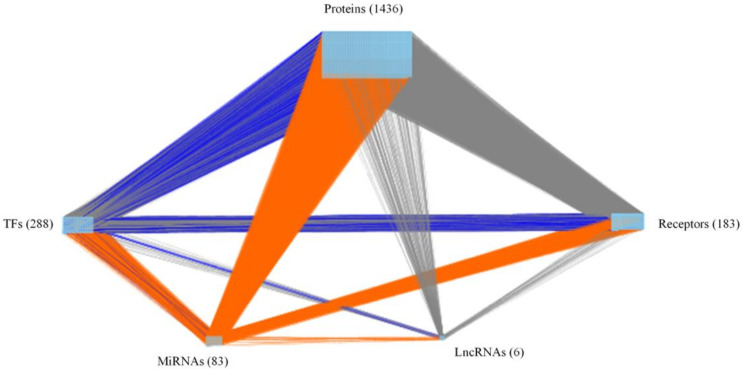
The core genome-wide genetic and epigenetic network (GWGEN) of TNBC. The grey lines indicate protein–protein interactions (PPIs); the blue lines denote transcriptional regulations by TFs and lncRNAs; and the orange lines represent post-transcriptional regulations by miRNAs. The numbers of receptors, proteins, TFs, miRNAs, and lncRNAs are 183, 1436, 288, 83, and 6, respectively.

**Figure 2:**
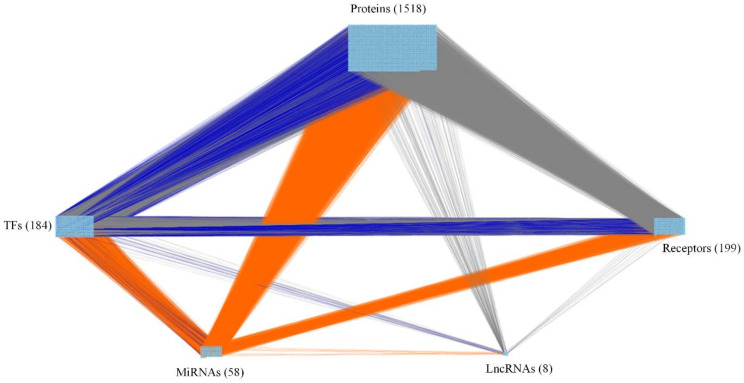
The core genome-wide genetic and epigenetic network (GWGEN) of non-TNBC. The grey lines indicate protein-protein interactions (PPIs); the blue lines denote transcriptional regulations by TFs and lncRNAs; and the orange lines represent post-transcriptional regulations by miRNAs. The numbers of receptors, proteins, TFs, miRNAs, and lncRNAs are 199, 1518, 184, 58, and 8, respectively.

**Figure 3:**
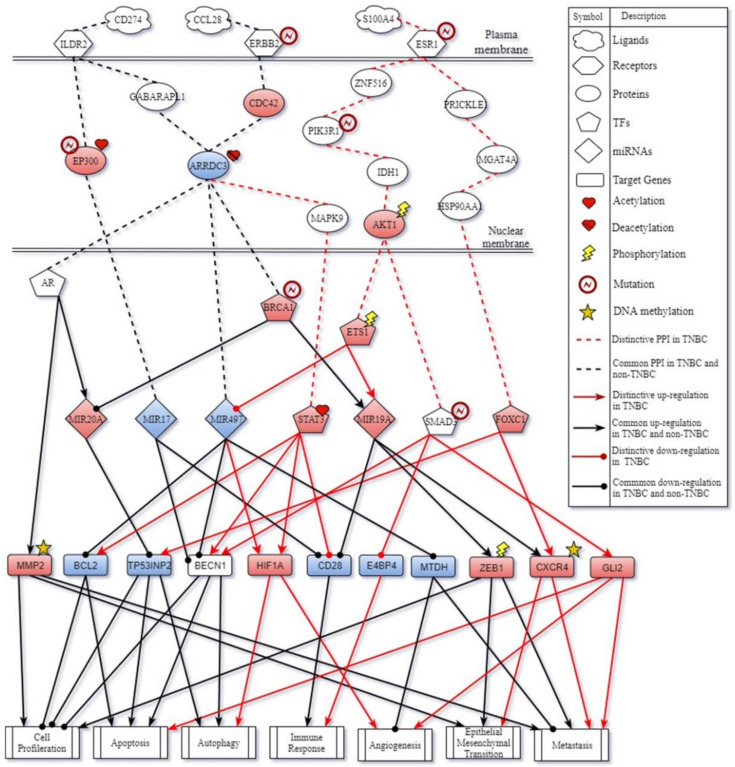
Core signaling pathways of triple negative breast cancer (TNBC). The red line indicates the specific core signaling pathway of TNBC; the black line indicates the common core signaling pathway of TNBC and non-TNBC; the black arrow head of the solid line means activation of cellular function; the black circle head of the solid line means inhibition of cellular function; the red node indicates high expression of protein, receptor, TF, miRNA, and target gene; and the blue node indicates low expression of protein, receptor, TF, miRNA, and target gene.

**Figure 4:**
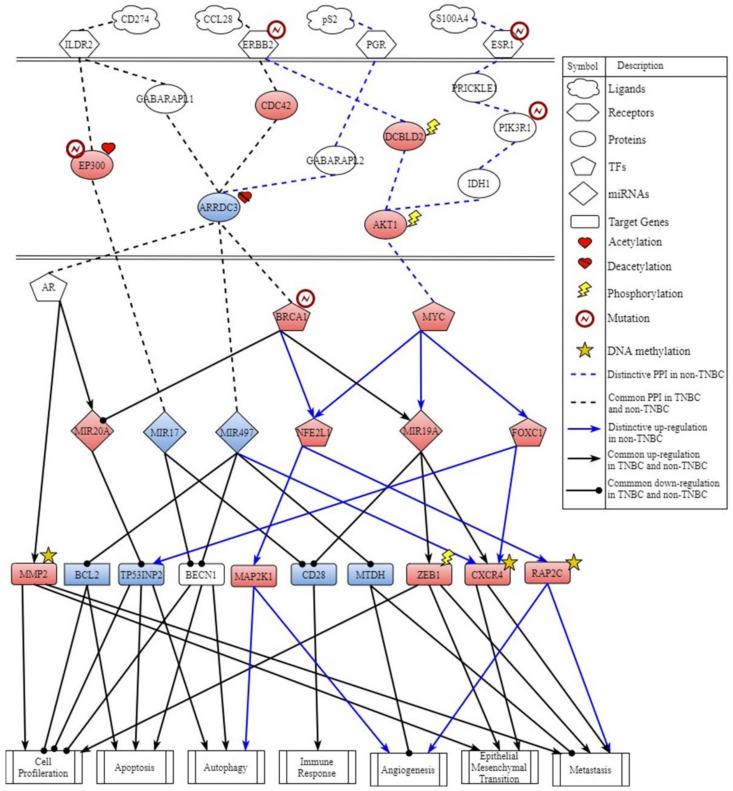
Core signaling pathways of non-triple negative breast cancer (non-TNBC). The black arrow head of the solid line means activation of cellular function; the black circle head of the solid line means inhibition of cellular function; the red node indicates high expression of protein, receptor, TF, miRNA, and target gene; the blue node indicates low expression of protein, receptor, TF, miRNA, and target gene; the blue line indicates distinctive core signaling pathway of non-TNBC; and the black line indicates common core signaling pathway of TNBC and non-TNBC.

**Figure 5:**
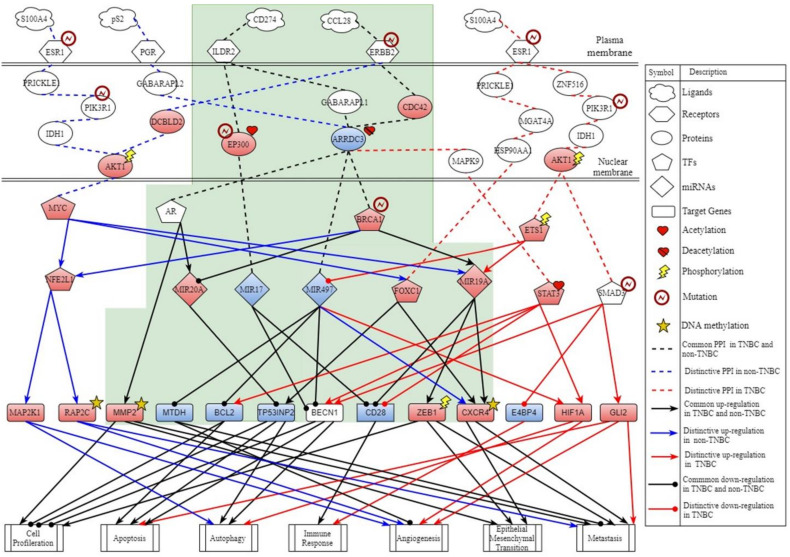
The common and distinctive core signaling pathways between triple negative breast cancer (TNBC) and non-triple negative breast cancer (non-TNBC). This figure summarizes the genetic and epigenetic progression mechanism of TNBC and non-TNBC. The core signaling pathways with the green background are the common core signaling pathways of TNBC and non-TNBC. The blue line indicates distinctive core signaling pathway of non-TNBC; the red line indicates distinctive core signaling pathway of TNBC; the black line indicates common core signaling pathway of TNBC and non-TNBC; the black arrow head of solid line means activation of cellular function; the black circle head of solid line means inhibition of cellular function; the red node indicates high expression of protein, receptor, TF, miRNA, and target gene; and the blue node indicates low expression of protein, receptor, TF, miRNA, and target gene.

By big database mining, we built a candidate GWGEN represented by a Boolean matrix (e.g., 0 or 1 if interaction is nonexistent or existent between two nodes). The candidate GWGEN is composed of candidate PPIN and candidate GRN. The TNBC and non-TNBC share the same candidate GWGEN. After doing systems modeling with the help of TNBC and non-TNBC microarray datasets, we evaluated system models’ parameters by system identification. Due to various experimental conditions, which might lead to error within data coming from different database, we used a system order detection scheme to prune false positives of regulations and interactions in candidate GWGEN for obtaining real GWGENs for TNBC and non-TNBC shown in Figures S1 and S2. The total number of nodes containing transcription factors (TFs), lncRNAs, miRNAs, receptors and proteins and edges of their interactions in candidate GWGEN and real GWGENs of TNBC and non-TNBC are in Table 1. It is noted that the nodes and edges decrease a lot comparing real GWGENs to candidate GWGEN. This phenomenon demonstrated that the false-positives caused by datasets coming from various experimental conditions were eliminated by system order detection approach. However, the real GWGENs of TNBC and non-TNBC are still too complicated to be analyzed. We utilized PNP method to extract the core GWGENs, which are shown in Figure 1 and Figure 2, from the real GWGENs. The higher the projection value is, the greater contribution would be made by the corresponding component in the real GWGENs. Here, we selected the top-ranked 2000 nodes with significant projection values that could reflect 85% of the real GWGENs to be our core GWGENs of TNBS and non-TNBC. Moreover, the enrichment analyses by The Database for Annotation, Visualization and Integrated Discovery (DAVID) showing related pathways of the core GWGENs in TNBC and non-TNBC are in Table 2 and Table 3, respectively. To be convenient for analyzing the distinctive and common pathogenesis of TNBC and non-TNBC, we denoted their core signaling pathways in Figure 3 and Figure 4 with respect to the KEGG pathways. The overlap of core signaling pathways of TNBC and non-TNBC, which are common core signaling pathways, could be found in Figure 5. In this study, first, we will discuss the distinctive core signaling pathways shown in Figure 3 and Figure 4 with red lines for TNBC and blues lines for non-TNBC, respectively. After that, we will discuss the common core signaling pathways shown in Figure 5 with black lines. For the common core signaling pathways, there are 58.8% overlap proteins and 63.6% overlap genes for TNBC; there are 70% overlap proteins and 70% overlap genes for non-TNBC. Considering the microenvironment and the epigenetic modifications which could be judged by the basal level term in system modeling, we investigated carcinogenic molecular mechanisms of TNBC and non-TNBC triggered by the ligands going through core signaling proteins, core TFs, and their corresponding core target genes in the nucleus. Based on our analytic results of the core signaling pathways, we could select two pools of essential biomarkers, which are *AKT1, BRCA1, FOXC1, MMP2, ETS1,* and *STAT3* for TNBC and *AKT1, BRCA1, FOXC1, MMP2,* and *NFE2L1* for non-TNBC. Consequently, we designed two filters considering regulation ability and toxicity with the help of CMap and the predicted values of LD50 to propose potential multi-molecule drugs for TNBC and non-TNBC, respectively.

### 2.1. Distinctive Core Signaling Pathways for TNBC

For the distinctive core signaling pathways of TNBC in the Figure 3 with red lines, receptor ERBB2, which was mutated in breast cancer, received microenvironment factor CCL28 to trigger TF STAT3 through signaling transduction proteins CDC42, ARRDC3, and MAPK9. The overexpression of TF STAT3 impacted by acetylation could not only upregulate target genes BCL2, BECN1, and HIF1A to induce autophagy, apoptosis, and angiogenesis and inhibit cell proliferation but also suppress CD28 to inhibit the immune response.

The next pathway, ESR1, which was mutated in breast cancer, received microenvironment factor S100A4 to regulate TF ETS1 and SMAD3 through signaling transduction proteins ZNF516, PIK3R1, IDH1, and AKT1. Both PIK3R1, modified by mutation, and AKT1, affected by phosphorylation, could deliver the signal from ESR1 to ETS1 and SMAD3 in TNBC. The overexpression of TF ETS1, which is abnormally activated, could upregulate miRNA MIR19A and downregulate miRNA MIR497. The overexpression of miRNA MIR19A awakened by an upstream signal could not only facilitate target genes ZEB1 and CXCR4, which were modified by phosphorylation and DNA methylation, respectively, to promote the cellular functions, including cell proliferation, EMT, and metastasis, but also downregulate target gene CD28 to deactivate the immune response. Inhibition of miRNA MIR497 caused the regulation of target genes BCL2 and HIF1A to induce autophagy, apoptosis, and angiogenesis. Moreover, mutation of TF SMAD3 could promote the cellular functions, such as autophagy, apoptosis, angiogenesis, and metastasis by the activation of target genes BECN1 and GLI2 and inhibit the immune response by the suppression of E4BP4.

On the cell membrane, once receptor ESR1 received ligand S100A4, it could also activate TF FOXC1 through signaling transduction proteins PRICKLE1, MGAT4A, and HSP90AA1. The overexpression of FOXC1 could upregulate target genes TP53INP2 and CXCR4 modified by DNA methylation to trigger autophagy, EMT, and metastasis. To summarize, the carcinogenic molecular mechanisms in TNBC result in the regulation of autophagy, inhibition of the immune response, preparation of angiogenesis, mediation of the progression of metastasis, and the survival of cancer cells in unfavorable microenvironment at the same time. Thus, based on carcinogenic molecular mechanisms triggered by the core signaling pathways of TNBC, we selected AKT1, ETS1, STAT3, and FOXC1 to be biomarkers for TNBC.

### 2.2. Distinctive Core Signaling Pathways for Non-TNBC

According to Figure 4, the distinctive core signaling pathways of non-TNBC, shown with blue lines, not only ligand CCL28 received by receptor ERBB2 but also ligand S100A4 received by receptor ESR1 could activate protein AKT1 to upregulate TF MYC by the signaling pathway derived through transduction protein DCBLD2 modified by the phosphorylation and the signaling pathway derived through PRICKLE1, PIK3R1, and IDH1, respectively. The overexpression of protein DCBLD2 and the mutation of protein PIK3R could help the process of signaling transduction. The overexpression of TF MYC activated by two signaling pathways upregulated downstream regulator NFE2L1, FOXC1, and MIR19A. The activation of TF NFE2L1 could regulate target genes BCL2, MAP2K1, and RAP2C to induce autophagy, angiogenesis, EMT, and metastasis. In these pathways, both BCL2 and RAP2C were modified by DNA methylation. Activation of miRNA MIR19A could not only positively regulate target genes ZEB1 and CXCR4, which were both modified by phosphorylation and DNA methylation, to promote the carcinogenic molecular mechanisms, including cell proliferation, EMT, and metastasis, but also negatively regulate target gene CD28 to suppress the immune response. The activation of FOXC1 upregulated target genes TP53INP2 and CXCR4, which were modified by DNA methylation, to trigger autophagy, EMT, and metastasis.

In the final core signaling pathway, receptor PGR received microenvironment factor pS2 and transmitted the signal through transduction proteins GABARAPL2 and ARRDC3 to TF BRCA1 and NFE2L1 activating target gene CXCR4 to stimulate EMT and metastasis in non-TNBC. We found that the core signaling pathways of non-TNBC lead to carcinogenic molecular mechanisms, including metastasis, angiogenesis, EMT, and cell proliferation. Thus, AKT1, NFE2L1, and FOXC1 were selected to be the biomarkers for non-TNBC.

### 2.3. Common Core Signaling Pathways of TNBC and Non-TNBC

Based on the projection values obtained from the PNP method, we investigated the common core signaling pathways between TNBC and non-TNBC shown in Figure 5 with black lines. The receptor ILDR2 received microenvironment factor CD274, known as PD-L1 (a regulation signaling of the immune response), to silence miRNA MIR17 through signaling transduction protein EP300 in TNBC and non-TNBC. The mutative protein EP300 in breast cancer, which was affected by acetylation, could promote the transmission of upstream signals to its downstream regulator. MiRNA MIR17 with low expression could negatively regulate target genes BECN1 and CD28 to promote the proliferation of cancer cells and inhibit autophagy, apoptosis, and the immune response. Moreover, receptor ILDR2 also regulated TF AR, TF BRCA1, and miRNA MIR497 via signaling transduction proteins GABAPAL1 and ARRDC3. Suppression of ARRDC3 expression in breast cancer cells involving the epigenetic silencing caused by the deacetylases directly impacted signaling to downstream TFs. TF AR not only activated target gene MMP2, which was modified by DNA methylation, to trigger cell proliferation, epithelial-mesenchymal transition (EMT), and metastasis, but also transmitted the signal from protein ARRDC3 to miRNA MIR20A. Mir20A silenced target gene TP53INP2 to inhibit autophagy, apoptosis, and promotes cell proliferation. MiRNA MIR497 with low expression silenced target genes BECN1, MTDH, and BCL2 to indulge cancer cell differentiation, angiogenesis, and metastasis.

For the next common core signaling pathway, the receptor ERBB2, which was mutated in breast cancer, received microenvironment factor CCL28 to activate TF BRCA1 through signaling transduction proteins CDC42 and ARRDC3 in TNBC and non-TNBC. Overexpression of protein CDC42 in breast cancer could induce the signaling to TF BRCA1. Mutative TF BRCA1 with abnormal overexpression in breast cancer could not only inhibit miRNA MIR20A to actuate target gene TP53INP2 to trigger autophagy and apoptosis, but also galvanize miRNA MIR19A to drive target genes CXCR4 and ZEB1, which were modified by DNA methylation and phosphorylation, respectively, to promote cell proliferation, angiogenesis, and metastasis. MiRNA MIR19A also downregulated the target gene CD28 to inhibit the immune response. In conclusion, the common core signaling pathways contribute to increasing genomic damage, escaping the immune checkpoint, promoting metastasis, enhancing the survival of cancer cells by carcinogenic molecular mechanisms, including autophagy, apoptosis, cell proliferation, immune response, angiogenesis, and metastasis in the extremely worst microenvironment. Therefore, we propose BRCA1 and MMP2 to be our common biomarkers in the TNBC and non-TNBC.

## 3. Discussion

### 3.1. The Carcinogenic Molecular Mechanisms in TNBC

In the core signaling pathways of TNBC, as shown in Figure 3 with red lines, ligand CCL28 binding to receptor ERBB2 transmits significant signals via transduction proteins CDC42, ARRDC3, and MAPK9 to upregulate TF STAT3, which not only facilitates autophagy, apoptosis, cell differentiation, angiogenesis, epithelial-mesenchymal transition (EMT), and metastasis, but also avoid the immune response in TNBC. It has been shown that TF STAT3 plays a crucial role of mediating tumor-induced immune suppression in various microenvironment conditions [29]. The overexpression of STAT3 modified by acetylation regulates target genes BECN1 and CD28 to trigger autophagy and the inhibition of the immune response. Here, in the core signaling pathway of TNBC, autophagy has the ability to regulate T-cell functions, which inhibit the immune response, and cell proliferation to reduce the consumption and accumulate abundant energy for angiogenesis.

In the next core signaling pathway in TNBC, ligand S100A4 binding to receptor ESR1 transmits signals through cascade proteins ZNF516, PIK3R1, IDH1, AKT1 to TF ETS1 and SMAD3, facilitating autophagy, cell differentiation, angiogenesis, epithelial-mesenchymal transition (EMT), metastasis, and the inhibition of the immune response. One study has shown that the mutation of ESR1 and ER frequently occurred in metastatic breast cancer, which would influence the response to hormone therapy [30]. Notably, the upstream signaling transmitted from receptor ESR1 results in the phosphorylation of protein AKT1 to activate TF ETS1 and SMAD3, triggering downstream carcinogenic molecular mechanisms. Moreover, TF ETS1 could activate target genes CXCR4 and ZEB1 to trigger metastasis mediated by downstream core signaling cascades through the suppression of tumor inhibitor, miRNA MIR497. On the other side, TF SMAD3 was identified to involve in cancer progression by regulating its target genes BECN1, GLI2, and E4BP4. Its target genes BECN1 would induce autophagy and E4BP4 suppressed by TF SMAD3 could promote cancer progression by reducing NK cell development [31]. Obviously, the activation of AKT1 modified by phosphorylation in the core signaling pathway leads to worse prognosis.

In the final core signaling pathway, the signaling transduction starting from ESR1 to TF FOXC1 through signaling transduction proteins PRICKLE1, MGAT4A, and HSP90AA1 could mediate carcinogenic molecular mechanisms containing autophagy and epithelial mesenchymal transition (EMT). Some literature suggested that TF FOXC1 plays an important role in tumor development and metastasis [32,33]. Clinical studies have also demonstrated that the elevated expression of FOXC1 was associated with poor prognosis in many kinds of cancers [34,35].

### 3.2. The Carcinogenic Molecular Mechanisms in Non-TNBC

In the core signaling pathways of non-TNBC, shown in Figure 4 with blue lines, ligand CCL28 binding to receptor ERBB2 and ligand S100A4 binding to ESR1 could transmit signaling through proteins DCBLD2, PRICKLE1, PIK3R1, IDH1, and AKT1 to upregulate the same TF MYC. The aberrant activation of receptor ERBB2 in human cancers promotes tumorigenesis through the stimulation of AKT signaling [36]. The overexpression of protein DCBLD2 modified by phosphorylation not only increases the expression level of AKT1, but also makes AKT1 modify by phosphorylation. This phenomenon activates TF MYC to interact with miRNA MIR19A and TFs NFE2L1 and FOXC1, regulating their corresponding target genes to trigger several carcinogenic molecular mechanisms, including autophagy, apoptosis, cell proliferation, angiogenesis, EMT, metastasis, and the inhibition of the immune response.

In the next core signaling pathway, ligand pS2 binding to receptor PGR passes signaling through signaling cascade proteins GABARAPL2 and ARRDC3 to TFs BRCA1 and NFE2L1. TF NFE2L1 interacting with mutative TF BRCA1 could upregulate target genes MAP2K1 and RAP2C to facilitate cell differentiation, angiogenesis, and metastasis. Moreover, the overexpression of TF NFE2L1 has been suggested to protect tumor cells by decreasing the toxicity of treatment [37]. Activated target gene RAP2C modified by DNA methylation could induce angiogenesis and metastasis to promote migration and invasion in non-TNBC [38].

### 3.3. The Common Carcinogenic Molecular Mechanisms between TNBC and Non-TNBC

In the first common core signaling pathway in both TNBC and non-TNBC, as shown in Figure 5 with black lines, microenvironment factor CD274, known as programmed death-ligand 1 (PD-L1), can not only simulate cancer cells proliferation, angiogenesis, epithelial-mesenchymal transition (EMT), and metastasis, but also inhibit autophagy, apoptosis, and the immune response through receptor ILDR2. The crucial signals are transmitted through signal transduction proteins EP300, GABARAPL1, and ARRDC3 to regulate TF AR, miRNA MIR20A, MIR17, and MIR497 in both TNBC and non-TNBC. Furthermore, the ligand PD-L1 is speculated to play a major role in suppressing the adaptive arm of immune system in many diseases. It is shown that the upregulation of PD-L1 may allow cancers to evade the host immune system [39]. According to a recent research, receptor ILDR2 has been identified as a novel B7-like protein with robust T cell inhibitory activity [40]. The role of transduction signaling protein EP300 mutated in cancer has been evidenced that it activated miRNA MIR17 to negatively regulate target genes BECN1 and CD28 to promote cell proliferation and escape the immune checkpoint [41,42,43]. Some studies have shown that protein ARRDC3 was implicated in tumor suppression by modulating the levels of carcinogenic genes [44,45]. In contrast, ARRDC3, with a low expression level, silenced by deacetylation, resulting in downstream regulators, could neither activate miRNA MIR497 to regulate target genes to inhibit tumor progression nor suppress TF AR and miRNA MIR20A to avoid cell proliferation and accumulation of genomic damage and instability [46]. Furthermore, target gene CD28 has been shown to be involved in immune checkpoint pathway [47]. The inhibition of target gene BECN1 attributes the loss of autophagy and apoptosis in this pathway [48,49,50]. Hence, this core signaling pathway worsens the conditions of patients with breast cancer via increasing genomic damage and escaping the immune checkpoint.

In the next common core signaling pathway, the microenvironment factor CCL28 binding to receptor ERBB2 starts to transmit through signaling transduction proteins CDC42 and ARRDC3 to TFs AR and BRCA1 and miRNA MIR497, facilitating autophagy, apoptosis, cell differentiation, angiogenesis, EMT, and metastasis, and inhibiting the immune response. Simultaneously, the TF BRCA1 with mutation regulates miRNAs MIR20A and MIR19A in both TNBC and non-TNBC. Furthermore, CCL28, mucosa-associated epithelial chemokine (MEC), has been suggested to have something to do with tumor progression and the involvement in inflammation [51,52]. Moreover, the mutation of receptor ERBB2 frequently appeared in breast cancer and participated in the migration of cancer cells [53,54,55]. The overexpression of protein CDC42 has been shown to enhance cell differentiation and metastasis [56]. The mutation of TF BRCA1 caused by the abnormal upstream signaling cascades coming from receptor ERBB2 can positively regulate target genes TP53INP2, ZEB1, and CXCR4, negatively regulate target gene CD28, and suppress miRNA MIR20A to induce autophagy, cell proliferation, and metastasis. It is worth noting that mutative TF BRCA1 could regulate target gene TP53INP2 to mediate autophagy and apoptosis simultaneously. Substantial evidences have demonstrated that autophagy, a double-edge sword on cancer development, was a tumor suppression mechanism, yet in most contexts, it facilitated tumorigenesis in stress [57,58,59].

### 3.4. Exploring Multi-Molecule Drugs for TNBC and Non-TNBC Based on Drug Regulation Ability and Toxicity

The systems biology approaches we proposed in this study helped us identify essential biomarkers with specific gene expression signature efficiently for TNBC and non-TNBC, respectively. Moreover, the biomarkers we found not only have higher projection values but also express abnormally. The goal of finding potential multi-molecule drugs is to explore compounds which could reverse the identified gene expression signature. In CMap, the connectivity score, which is in the range of −1 to 1, reflects the closeness or connection between the expression profiles. A positive correlation denotes the degree of similarity and a negative correlation emphasizes an inverse similarity between a query signature and an individual chemical perturbation. Therefore, if the selected biomarkers (drug targets) have an abnormal upregulated gene signature, we would choose the drugs with negative correlation for the reversed mapping. In contrast, if we found that the selected biomarkers (drug targets) are downregulated abnormally, the drugs with positive correlation would be considered. Moreover, we also provide the predicted LD50 computed by admetSAT tool [60], as reference for each potential compound.

In Table S1, with the help of proposed systems medicine design procedure, the top candidate drugs for each drug target are shown. Notably, resveratrol is identified to be a potential drug for five drug targets, which include BRCA1, ETS1, FOXC1, STAT3, and NFE2L1. Resveratrol is a nutraceutical drug with several therapeutic effects. It is thought to act as a chemopreventative agent by attenuating autophagy, cell growth, and proliferation, which are associated with cancer initiation and progression [61]. In addition, patients often occur drug resistance due to the activation of the oncogenic Akt signaling and the upregulation of autophagy, which protects cancer cells from apoptosis. There was research demonstrating that the combination therapy of rapamycin together with resveratrol maintains the inhibition of mTORC1 signaling, which could prevent the upregulation of Akt activation and autophagy to induce the apoptosis of breast cancer cells [62]. Interestingly, rapamycin, known as sirolimus, is also identified to be a potential drug for targets, such as AKT1, ETS1, STAT3, and NFE2L1. Sirolimus is a promising therapeutic agent with both immunosuppressant and anti-tumor properties [63]. It could inhibit the translation of critical mRNAs that are involved in the cell cycle progression and cell proliferation, which are hallmarks of carcinogenesis [64]. Moreover, both carbamazepine and prednisolone are identified to be potential drugs for four targets, including FOXC1, MMP2, ETS1, and STAT3. A phase II trial has demonstrated that prednisone treatment was advantageous for breast cancer [65]. Carbamazepine (CBZ) is a well-known anti-epileptic drug that has been used in clinical practice for more than four decades. However, a recent study revealed that CBZ has similar function to histone deacetylase (HDAC) inhibitor, which has been confirmed as an anti-cancer drug [66]. One study has shown that CBZ could synergize with trastuzumab to further downregulate Her-2 protein and inhibit breast cancer cell proliferation [67]. Furthermore, verapamil is identified to be a potential drug for targets FOXC1 and NFE2L1. It is capable of suppressing tumor progression by the inhibition of tumor cell growth and metastasis, enhancement of tumor apoptosis, and reduction in microvascular density [68]. In summary, by the proposed systems medicine design procedure, we suggested two multi-molecule drugs: resveratrol, sirolimus, and prednisolone for TNBC and resveratrol, sirolimus, carbamazepine, and verapamil for non-TNBC, which are shown in Table 4 and Table 5.

## 4. Materials and Methods

### 4.1. Overview of the Systems Medicine Design Procedure

**Figure 6:**
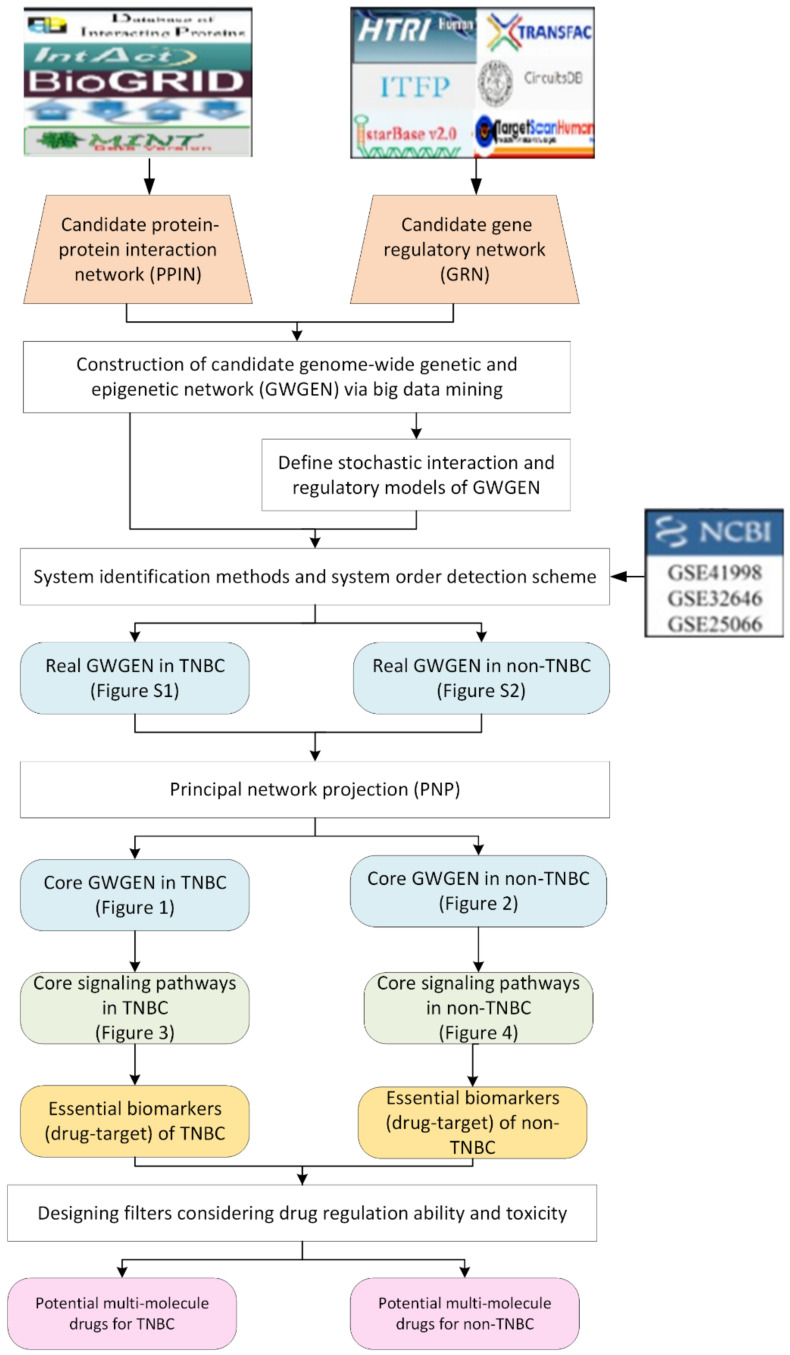
Flowchart of systems medicine procedure for finding essential biomarkers (drug targets) and proposing potential multi-molecule drugs for TNBC and non-TNBC.

To find the essential biomarkers based on carcinogenic molecular mechanisms and propose potential multi-molecule drugs for TNBC and non-TNBC, a flowchart is given in Figure 6. In the viewpoint of the systems biology approaches, we could separate our systems medicine design procedure into five steps: (1) Using big database mining technique to construct a candidate GWGEN consisting of candidate PPIN and GRN; (2) Doing system modeling on proteins, genes, miRNAs, and lncRNAs; (3) Applying systems identification and systems order detection approaches assisting with the microarray data of TNBC and non-TNNC to obtain real GWGENs; (4) Applying the principal network projection method on the real GWGENs to get the core GWGENs and core signaling pathways denoted by KEGG pathways; (5) Designing filters of drug regulation ability and toxicity to obtain potential multi-molecule drugs. The detailed information of each step in Figure 6 are elucidated in the following section.

### 4.2. Construction Candidate Genome-Wide Genetic and Epigenetic Network (GWGEN) by Microarray Data of TNBC and Non-TNBC

In this study, we used three microarray datasets of breast cancer with accession numbers GSE41998, GSE32646, and GSE25066, obtained from the NCBI gene expression omnibus (GEO) [69,70,71]. Their corresponding platforms are GPL571, GPL570, and GPL96, respectively. There were 284 samples for the TNBC and 544 samples for the non-TNBC. To avoid the overfitting problem in the network construction, the maximum degree of each component in the PPIN and GRN should be less than the sample number. The candidate GWGEN consists of candidate PPIN and candidate GRN, which were represented in a Boolean matrix, respectively. If there was an interaction between two elements, we would give one; if there was no interaction, we would give zero on their corresponding position. With the big database mining, for the candidate PPIN, having protein–protein interactions (PPIs), information was mined from DIP [72], IntAct [73], BioGRID [74], BIND [75], and MINT [76]; the candidate GRN containing the interaction information within the TFs to genes, lncRNAs, and miRNAs was available at HTRIdb [77], IFTP [78], TargetScan [79], and CircuirtsDB 2 [80].

### 4.3. Systems Modeling for Candidate Genome-Wide Genetic and Epigenetic Network (GWGEN)

In order to identify the real GWGENs for TNBC and non-TNBC, we have to formulate proteins, genes, miRNAs, and lncRNAs considering the basal level and stochastic noise caused by model residue and data measurement noise. For the protein interaction model, the *i*-th protein for sample *n* is given in the following equation:

(1) $$\begin{array}{l} p_{i}\left[n\right]=\sum_{\begin{array}{l} j=1\\ j\neq i \end{array}}^{I_{i}}\lambda_{ij}p_{i}\left[n\right]p_{j}\left[n\right]+\psi_{i,PPIs}+\theta_{i,PPI}\left[n\right],\;\\ \mathrm{for}\;i=1,\ldots ,I,\;n=1,\ldots ,N \end{array}$$

where $\lambda_{ij}$ denotes the interaction ability between the *i-*th protein and the *j-*th protein; $p_{i}\left[n\right]$ and $p_{j}\left[n\right]$ represent the expression level of the *i*-th and *j*-th protein for the sample n; $I_{i}$ indicates the total number of proteins interacting with the *i-*th protein; I is the total number of proteins in candidate PPIN; N is the total number of samples (patient); $\psi_{i,PPI}$ represents the basal level of the *i-*th protein expression; and $\theta_{i,PPI}\left[n\right]$ is the stochastic noise of the *i-*th protein for the sample *n* caused by model uncertainty and data measurement noise.

For the gene regulation model, the *x-*th gene in sample *n* could be described by the following equation:

(2) $$\begin{array}{l} g_{x}\left[n\right]=\sum_{\begin{array}{l} u=1\\ u\neq x \end{array}}^{U_{x}}\alpha_{xu}t_{u}\left[n\right]+\sum_{k=1}^{K_{x}}\beta_{xk}l_{k}\left[n\right]-\sum_{v=1}^{V_{x}}\gamma_{xv}m_{v}\left[n\right]g_{x}\left[n\right]+\psi_{x}+\theta_{x}\left[n\right],\;\\ \mathrm{for}\;x=1,\ldots ,X,\;n=1,\ldots ,N \end{array}$$

where $g_{x}\left[n\right]$ represents the expression level of the *x-*th gene; $U_{x}$ indicates the total number of TFs binding to the *x*-th gene; $K_{x}$ denotes the total number of lncRNAs binding to the *x*-th gene; $V_{x}$ is the total number of miRNAs inhibiting the *x*-th gene; $\alpha_{xu}$ and $\beta_{xk}$ denote the transcription regulatory ability of the *u-*th TF and the *k-*th lncRNA on the x-th gene; $\gamma_{xv}$, which is larger than zero and ($\gamma_{xv}\geq 0$) is the post-transcriptional regulation ability inhibiting the *x*-th gene; $t_{u}\left[n\right]$, $l_{k}\left[n\right]$, and $m_{v}\left[n\right]$ indicate the expression level of the *u-*th TF, the *k-*th lncRNA, and *v-*th miRNA, respectively; X is the total number of genes; N is the total number of samples (patients); $\psi_{x}$ represents the basal level of the *x-*th gene expression; and $\theta_{x}\left[n\right]$ is the stochastic noise including model uncertainty and data noise. The same concept of systems modeling on miRNAs and lncRNAs are shown in Supplementary Material 1.1.

### 4.4. Systems Identification and Systems Model Order Selection for Obtaining Real GWGEN of TNBC and Non-TNBC

In the previous section, we have described systems modeling for proteins, genes, miRNAs, and lncRNAs in the candidate GWGEN. In order to identify parameters in the candidate PPIN and candidate GRN, we have to solve constraint least-square problems. Here, we rewrite PPI interactive Equation (1) in the linear regression form as below:

(3) $$\begin{array}{l} p_{i}[n]=\left[p_{i}[n]p_{1}[n]\cdots p_{i}[n]p_{I_{i}}[n]1\right]\times \left[\begin{array}{c} \lambda_{i1}\\ \vdots \\ \lambda_{iI_{i}}\\ \psi_{i} \end{array}\right]+\theta_{i}\left[n\right]\\ =\;\Phi_{i,P}[n]\cdot \omega_{i,P}+\theta_{i}\left[n\right],\;\mathrm{for}\;i=1,\ldots ,I. \end{array}$$

where $\Phi_{i,p}[n]$ denotes the regression vector which can be obtained from the microarray data and $\theta_{i}[n]$ represents the unknown parameter vector for the *i-*th protein in PPIN. Equation (3) of the *i*-th protein can be augmented for *N* samples, shown in the following:

(4) $$\left[\begin{array}{c} \;p_{i}[1]\\ \;p_{i}[2]\\ \vdots \\ p_{i}[N] \end{array}\right]=\left[\begin{array}{c} \Phi_{i,P}[1]\\ \Phi_{i,P}[2]\\ \vdots \\ \Phi_{i,P}[N] \end{array}\right]\cdot \omega_{i,P}+\left[\begin{array}{c} \theta_{i}\left[1\right]\\ \theta_{i}\left[2\right]\\ \vdots \\ \theta_{i}\left[N\right] \end{array}\right],\;\mathrm{for}\;i=1,\ldots ,I,\;n=1,\ldots N.$$

Equation (4) could be simply shown as:

(5) $$P_{i}=\Omega_{i,P}\cdot \omega_{i,P}+\vartheta_{i}$$

where:

(6) $$P_{i}=\left[\begin{array}{c} \;p_{i}[1]\\ \;p_{i}[2]\\ \vdots \\ p_{i}[N] \end{array}\right],\;\Omega_{i,p}=\left[\begin{array}{c} \Phi_{i,P}[1]\\ \Phi_{i,P}[2]\\ \vdots \\ \Phi_{i,P}[N] \end{array}\right],\;\vartheta_{i}=\left[\begin{array}{c} \theta_{i}\left[1\right]\\ \theta_{i}\left[2\right]\\ \vdots \\ \theta_{i}\left[N\right] \end{array}\right]$$

Therefore, the estimated vector ${\hat{\omega }}_{i,p}$ can be obtained by solving the following linear least-square problem via MATLAB optimization toolbox:

(7) $${\hat{\omega }}_{i,P}=\underset{\omega_{i,P}}{\min}\frac{1}{2}{\left\|\Omega_{i,P}\cdot \omega_{i,P}-P_{i}\right\|}_{2}^{2}$$

The linear regression form of the gene regulatory Equation (2) in GRN could be described as below:

(8) $$\begin{array}{l} g_{x}[n]=\left[t_{1}[n]\cdots {\;t}_{U_{x}}[n]l_{1}[n]\cdots l_{K_{x}}[n]g_{x}[n]m_{1}[n]\cdots g_{x}[n]m_{V_{x}}[n]1\right]\times \left[\begin{array}{c} \alpha_{x1}\\ \vdots \\ \alpha_{xU_{x}}\\ \beta_{x1}\\ \vdots \\ \beta_{xK_{x}}\\ -\gamma_{x1}\\ \vdots \\ -\gamma_{xV_{x}}\\ \;\psi_{x} \end{array}\right]+\theta_{x}\left[n\right]\\ \;=\;\Phi_{x,G}[n]\cdot \omega_{x,G}+\theta_{x}\left[n\right],\;\mathrm{for}\;x=1,\ldots ,X. \end{array}$$

where $\Phi_{x,G}[n]$ indicates the regression vector, which can be obtained from the microarray data, and $\omega_{x,G}$ represents the unknown parameter vector for the *x-*th gene in GRN. Equation (8) of the *x-*th gene could be augmented for *N* samples in the following form:

(9) $$\left[\begin{array}{c} g_{x}[1]\\ g_{x}[2]\\ \vdots \\ g_{x}[N] \end{array}\right]=\left[\begin{array}{c} \Phi_{x,G}[1]\\ \Phi_{x,G}[2]\\ \vdots \\ \Phi_{x,G}[N] \end{array}\right]\cdot \omega_{x,G}+\left[\begin{array}{c} \theta_{x}\left[1\right]\\ \theta_{x}\left[2\right]\\ \vdots \\ \theta_{x}\left[N\right] \end{array}\right],\;x=1,\ldots ,X,\;n=1,\ldots ,N.$$

Equation (9) could be simply described as:

(10) $$G_{x}=\Omega_{x,G}\cdot \omega_{x,G}+\vartheta_{x}$$

where:

(11) $$G_{x}=\left[\begin{array}{c} g_{x}[1]\\ g_{x}[2]\\ \vdots \\ g_{x}[N] \end{array}\right],\;\Omega_{x,G}=\left[\begin{array}{c} \Phi_{x,G}[1]\\ \Phi_{x,G}[2]\\ \vdots \\ \Phi_{x,G}[N] \end{array}\right],\;\vartheta_{x}=\left[\begin{array}{c} \theta_{x}\left[1\right]\\ \theta_{x}\left[2\right]\\ \vdots \\ \theta_{x}\left[N\right] \end{array}\right]\;$$

Hence, by solving the constrained linear least-square problem in (12), the estimated vector ${\hat{\omega }}_{x,G}$ could be obtained via MATLAB optimization toolbox. Moreover, the miRNA repression parameters $-\gamma_{x,v}$ are guaranteed to be non-positive, i.e., $-\gamma_{x,v}\leq 0$ for $v=1,\ldots ,V_{x}$.

(12) $$\begin{array}{l} {\hat{\omega }}_{x,G}\;=\;\underset{\omega_{x,G}}{\min}\frac{1}{2}{\left\|\Omega_{x,G}\cdot \omega_{x,G}-G_{x}\right\|}_{2}^{2},\\ \mathrm{subject}\;\mathrm{to}\;\left[\begin{array}{ccccccccccccc} 0 & \cdots & \cdots & 0 & 0 & \cdots & \cdots & 0 & 1 & 0 & \cdots & 0 & 0\\ \vdots & \ddots & & \vdots & \vdots & \ddots & & \vdots & 0 & \ddots & \ddots & \vdots & \vdots \\ \vdots & & \ddots & \vdots & \vdots & & \ddots & \vdots & \vdots & \ddots & \ddots & 0 & \vdots \\ 0 & \cdots & \cdots & 0 & 0 & \cdots & \cdots & 0 & 0 & \cdots & 0 & 1 & 0 \end{array}\right]\omega_{x,G}\leq \left[\begin{array}{c} 0\\ \begin{array}{l} \vdots \\ \vdots \end{array}\\ 0 \end{array}\right].\\ \;\;\;\;\;\;\;\;\;\;\;\;\;\;\;\;\;\;\;\;\;\;\;\;\;\;\;\;\;\;\;\;\;\;\;\;\;\;U_{x}\;\;\;\;\;\;\;\;\;\;\;\;\;\;\;\;\;\;\;\;\;\;\;\;\;\;\;\;\;\;K_{x}\;\;\;\;\;\;\;\;\;\;\;\;\;\;\;\;\;\;\;\;\;\;\;\;\;\;\;\;V_{x} \end{array}$$

As a matter of fact, various experimental conditions might lead to error within data coming from different databases; thus, we applied the Akaike Information Criterion (AIC) to help us prune the false positives and detect the system order of real GWGENs. For the PPI interaction model in (5), we used AIC to detect the number of interactions of the *i*-th protein. The corresponding equation is defined as below:

(13) $$\begin{array}{l} AIC(I_{i})=\log({\hat{\varepsilon }}_{i,P}^{2})+\frac{2(\Delta_{i,P})}{N},\\ \mathrm{where}\;{\hat{\varepsilon }}_{i,P}=\sqrt{\frac{\left(P_{i}-{\left(\Omega_{i,P}\cdot {\hat{\omega }}_{i,P}\right)}^{T}(P_{i}-(\Omega_{i,P}\cdot {\hat{\omega }}_{i,P})\right)}{N}},\Delta_{i,P}=I_{i}+1. \end{array}$$

where ${\hat{\varepsilon }}_{i,P}^{2}$ and $\Delta_{i,P}$ represent the estimated residual error and number (order) of parameters of the *i-*th protein in (13) of the PPIN and ${\hat{\omega }}_{i,P}$ denotes the estimated vector of the *i-*th protein in (7). According to the AIC theory, the real system order $I_{i}^{*}$ would minimize $AIC(I_{i}^{*})$. The insignificant protein interactions, which are out of real system order $I_{i}^{*}$, would be pruned away to get the real PPI interaction model.

The AIC equation for the gene regulatory model is defined as below:

(14) $$\begin{array}{l} AIC(U_{x},K_{x},V_{x})=\log({\hat{\varepsilon }}_{x,G}^{2})+\frac{2(\Delta_{x,G})}{N},\\ \mathrm{were}\;{\hat{\varepsilon }}_{x,G}=\sqrt{\frac{\left(G_{x}-{\left(\Omega_{x,G}\cdot {\hat{\omega }}_{x,G}\right)}^{T}(G_{x}-(\Omega_{x,G}\cdot {\hat{\omega }}_{x,G})\right)}{N}},\Delta_{x,G}=U_{x}+K_{x}+V_{x}+1. \end{array}$$

where ${\hat{\varepsilon }}_{x,G}^{2}$ and $\Delta_{x,G}$ represent the estimated residual error and the number (order) of parameters for the *x-*th gene in the GRN, respectively and ${\hat{\omega }}_{x,G}$ is the estimated vector of the *x-*th gene in (12). The real system order $U_{x}^{*},K_{x}^{*},V_{x}^{*}$ would minimize $AIC(U_{x}{}^{*},K_{x}{}^{*},V_{x}{}^{*})$. We apply the same system identification and system model selection approaches on the miRNAs regulatory model and lncRNAs regulatory model, shown in Supplementary Materials 1.2. Based on the methods mentioned above, we obtain real GEGWNs for TNBC and non-TNBC, which are shown in Figures S1 and S2 of the Supplementary Materials.

### 4.5. Principal Network Projection (PNP) Method to Extract Core GWGENs of TNBC and Non-TNBC from Their Corresponding Real GWGENs

The real GWGENs of TNBC and non-TNBC are still too complicated, and it is not easy to further investigate their carcinogenic molecular mechanisms directly. In order to extract the core GWGENs from the real GWGENs, we propose the principal network projection (PNP) method. To prepare for applying PNP method to extract the core network, we have to construct a combined network matrix W that contains all the estimated parameters in the real GWGENs as follows:

(15) $$W=\left[\begin{array}{ccc} w_{protein↔protein} & 0 & 0\\ w_{TF\to gene} & w_{lncRNA\to gene} & w_{miRNA\to gene}\\ w_{TF\to lncRNA} & w_{lncRNA\to }{}_{lncRNA} & w_{miRNA\to lncRNA}\\ w_{TF\to miRNA} & w_{lncRNA\to }{}_{miRNA} & w_{miRNA\to miRNA} \end{array}\right]$$

where the sub-network matrix $w_{protein↔protein}$ consists of interaction abilities of proteins; the sub-network matrices $w_{TF\to gene}$, $w_{lncRNA\to gene}$, and $w_{miRNA\to gene}$ contain the estimated TFs transcriptional regulatory abilities, lncRNAs transcriptional regulatory abilities, and miRNAs post-transcriptional regulatory abilities on genes, respectively; the sub-network matrices $w_{TF\to lncRNA}$, $w_{lncRNA\to }{}_{lncRNA}$, and $w_{miRNA\to lncRNA}$ include the estimated TFs transcriptional regulatory abilities, lncRNAs transcriptional regulatory abilities, and miRNAs post-transcriptional regulatory abilities on lncRNAs, respectively; and the sub-network matrices $w_{TF\to miRNA}$, $w_{lncRNA\to }{}_{miRNA}$, and $w_{miRNA\to miRNA}$ composed of the estimated TFs transcriptional regulatory abilities, lncRNAs transcriptional regulatory abilities, and miRNAs post-transcriptional regulatory abilities on miRNAs, respectively. The combined network matrix is given in the following:

(16) $$W=\left[\begin{array}{ccccccccccccccc} {\hat{\lambda }}_{11} & \cdots & {\hat{\lambda }}_{1j} & \cdots & {\hat{\lambda }}_{1I} & 0 & \cdots & 0 & \cdots & 0 & 0 & \cdots & 0 & \cdots & 0\\ \vdots & \ddots & \vdots & \ddots & \vdots & \vdots & \ddots & \vdots & \ddots & \vdots & \vdots & \ddots & \vdots & \ddots & \vdots \\ {\hat{\lambda }}_{i1} & \cdots & {\hat{\lambda }}_{ij} & \cdots & {\hat{\lambda }}_{iI} & 0 & \cdots & 0 & \cdots & 0 & 0 & \cdots & 0 & \cdots & 0\\ \vdots & \ddots & \vdots & \ddots & \vdots & \vdots & \ddots & \vdots & \ddots & \vdots & \vdots & \ddots & \vdots & \ddots & \vdots \\ {\hat{\lambda }}_{I1} & \cdots & {\hat{\lambda }}_{Ij} & \cdots & {\hat{\lambda }}_{II} & 0 & \cdots & 0 & \cdots & 0 & 0 & \cdots & 0 & \cdots & 0\\ {\hat{\alpha }}_{11} & \cdots & {\hat{\alpha }}_{1u} & \cdots & {\hat{\alpha }}_{1U} & {\hat{\beta }}_{11} & \cdots & {\hat{\beta }}_{1k} & \cdots & {\hat{\beta }}_{1K} & -{\hat{\gamma }}_{11} & \cdots & -{\hat{\gamma }}_{1v} & \cdots & -{\hat{\gamma }}_{1V}\\ \vdots & \ddots & \vdots & \ddots & \vdots & \vdots & \ddots & \vdots & \ddots & \vdots & \vdots & \ddots & \vdots & \ddots & \vdots \\ {\hat{\alpha }}_{x1} & \cdots & {\hat{\alpha }}_{xu} & \cdots & {\hat{\alpha }}_{xU} & {\hat{\beta }}_{x1} & \cdots & {\hat{\beta }}_{xk} & \cdots & {\hat{\beta }}_{xK} & -{\hat{\gamma }}_{x1} & \cdots & -{\hat{\gamma }}_{xv} & \cdots & -{\hat{\gamma }}_{xV}\\ \vdots & \ddots & \vdots & \ddots & \vdots & \vdots & \ddots & \vdots & \ddots & \vdots & \vdots & \ddots & \vdots & \ddots & \vdots \\ {\hat{\alpha }}_{X1} & \cdots & {\hat{\alpha }}_{Xu} & \cdots & {\hat{\alpha }}_{XU} & {\hat{\beta }}_{X1} & \cdots & {\hat{\beta }}_{Xk} & \cdots & {\hat{\beta }}_{XK} & -\gamma_{X1} & \cdots & -{\hat{\gamma }}_{Xv} & \cdots & -{\hat{\gamma }}_{XV}\\ {\hat{\delta }}_{11} & \cdots & {\hat{\delta }}_{1u} & \cdots & {\hat{\delta }}_{1U} & {\hat{\Gamma }}_{11} & \cdots & {\hat{\Gamma }}_{1k} & \cdots & {\hat{\Gamma }}_{1K} & -{\hat{\tau }}_{11} & \cdots & -{\hat{\tau }}_{1v} & \cdots & -{\hat{\tau }}_{1V}\\ \vdots & \ddots & \vdots & \ddots & \vdots & \vdots & \ddots & \vdots & \ddots & \vdots & \vdots & \ddots & \vdots & \ddots & \vdots \\ {\hat{\delta }}_{y1} & \cdots & {\hat{\delta }}_{yu} & \cdots & {\hat{\delta }}_{yU} & {\hat{\Gamma }}_{y1} & \cdots & {\hat{\Gamma }}_{yk} & \cdots & {\hat{\Gamma }}_{yK} & -{\hat{\tau }}_{y1} & \cdots & -{\hat{\tau }}_{yv} & \cdots & -{\hat{\tau }}_{yV}\\ \vdots & \ddots & \vdots & \ddots & \vdots & \vdots & \ddots & \vdots & \ddots & \vdots & \vdots & \ddots & \vdots & \ddots & \vdots \\ {\hat{\delta }}_{Y1} & \cdots & {\hat{\delta }}_{Yu} & \cdots & {\hat{\delta }}_{YU} & {\hat{\Gamma }}_{Y1} & \cdots & {\hat{\Gamma }}_{Yk} & \cdots & {\hat{\Gamma }}_{YK} & -{\hat{\tau }}_{Y1} & \cdots & -{\hat{\tau }}_{Yv} & \cdots & -{\hat{\tau }}_{YV}\\ {\hat{\upsilon }}_{11} & \cdots & {\hat{\upsilon }}_{1u} & \cdots & {\hat{\upsilon }}_{1U} & {\hat{\sigma }}_{11} & \cdots & {\hat{\sigma }}_{1k} & \cdots & {\hat{\sigma }}_{1K} & -{\hat{\zeta }}_{11} & \cdots & -{\hat{\zeta }}_{1k} & \cdots & -{\hat{\zeta }}_{1K}\\ \vdots & \ddots & \vdots & \ddots & \vdots & \vdots & \ddots & \vdots & \ddots & \vdots & \vdots & \ddots & \vdots & \ddots & \vdots \\ {\hat{\upsilon }}_{z1} & \cdots & {\hat{\upsilon }}_{zu} & \cdots & {\hat{\upsilon }}_{zU} & {\hat{\sigma }}_{z1} & \cdots & {\hat{\sigma }}_{zk} & \cdots & {\hat{\sigma }}_{zK} & -{\hat{\zeta }}_{z1} & \cdots & -{\hat{\zeta }}_{zk} & \cdots & -{\hat{\zeta }}_{zK}\\ \vdots & \ddots & \vdots & \ddots & \vdots & \vdots & \ddots & \vdots & \ddots & \vdots & \vdots & \ddots & \vdots & \ddots & \vdots \\ {\hat{\upsilon }}_{Z1} & \cdots & {\hat{\upsilon }}_{Zu} & \cdots & {\hat{\upsilon }}_{ZU} & {\hat{\sigma }}_{Z1} & \cdots & {\hat{\sigma }}_{Zk} & \cdots & {\hat{\sigma }}_{ZK} & -{\hat{\zeta }}_{Z1} & \cdots & -{\hat{\zeta }}_{Zk} & \cdots & -{\hat{\zeta }}_{ZK} \end{array}\right]\in {ℜ}^{\left(I*+X*+Y*+Z*)\times (U*+K*+V*\right)}$$

The PNP method is based on singular value decomposition of W in (16) as follows:

(17) $$W=TDH^{T}$$

where $T\in {ℜ}^{\left(I*+X*+Y*+Z*)\times (U*+K*+V*\right)}$ and $H\in {ℜ}^{\left(I*+X*+Y*+Z*)\times (U*+K*+V*\right)}$ are the unitary matrix and $D=diag(d_{1},\cdots ,d_{s},\cdots ,d_{U+K+V})\in {ℜ}^{\left(U*+K*+V*)\times (U*+K*+V*\right)}$ represents the diagonal matrix, which is composed of U*+K*+V* singular values of W in descending order (i.e., $d_{1}\geq \cdots \geq d_{s}\geq \cdots \geq d_{U+K+V}\geq 0$).

The fraction of eigenexpression $\left\{E_{s}\right\}$ is calculated from the eigenexpression levels $\left\{d_{s}\right\}$ which are listed in the diagonal of D. The eigenexpression fraction $E_{s}$ is defined as below:

(18) $$E_{s}=\frac{d_{s}^{2}}{\sum_{s=1}^{U+K+V}d_{s}^{2}}$$

In terms of energy, we chose minimum *S* singular vectors of T and H with energy (i.e., $\sum_{m=1}^{S}E_{s}$) satisfying $\sum_{m=1}^{S}E_{s}\geq 0.85$. The top *S* principal singular vectors construct 85% of the principal network structure of the GWGEN. Next, here we define the projection of W to the top *S* right singular vectors of H for extracting important downstream nodes in the following:

(19) $$\begin{array}{l} H(c,r)=h^{T}{}_{r,:}\cdot w_{:,c}\;,\;\\ \mathrm{for}\;c=1,\ldots (U+K+V),\;r=1,\ldots S. \end{array}$$

where $w_{:,c}$ is the *c-*th column vector of W and $h_{r,:}^{T}$ is the *r-*th row vector of $H^{T}$. Afterward, we defined the 2-norm projection value of each downstream node in the real GWGENs to the top S right-singular vectors, which stands for 85% of the principal network structure as follows:

(20) $$\begin{array}{l} Q(c)=\sqrt{\sum_{r=1}^{S}H^{2}(c,r)}\;,\\ \mathrm{for}\;c=1,\ldots (U+K+V),\;r=1,\ldots S. \end{array}$$

where Q(c) is the 2-norm projection value of each downstream *c-*th node on the top *S* right-singular vectors. If the projection value *Q*(*c*) is close to zero, the corresponding *c*-th node is insignificant and nearly independent to the principal network structure. Conversely, the larger the projection value of a node in real GWGEN, the higher the possibility that the node is an essential component of the principal network structure. Similarly, we could use the same method to calculate the projection values of the upstream nodes through taking each row vector of combined network matrix W to project on the top *S* left-singular vectors. In conclusion, the core GWGENs of TNBC and non-TNBC shown in Figure 1 and Figure 2 could be extracted from the real GWGENs based on the projection values of the downstream nodes and upstream nodes.

## Figures and Tables

**Table 1:** The total number of nodes and edges in candidate GWGENs and identified real GWGENs of TNBC and non-TNBC.

Nodes/Edges	Candidate GWGEN	Real TNBC GWGEN	Real Non-TNBC GWGEN
TF-lncRNA	375	271	276
TF-miRNA	526	500	503
TF-protein	85,782	80,065	79,471
TF-TF	32,600	26,025	25,514
TFs	2567	2033	2108
lncRNA-lncRNA	6	5	5
lncRNA-miRNA	0	0	0
lncRNA-protein	1036	590	717
lncRNA-TF	420	184	220
lncRNAs	425	313	238
miRNA-lncRNA	88	61	64
miRNA-miRNA	1	1	1
miRNA-protein	31,020	20,206	20,861
miRNA-TF	6708	3747	3551
miRNAs	205	143	143
Receptors	2377	2207	2211
PPIs	4,639,077	2,478,528	1,967,333
Proteins	15,361	14,993	15,282
Total nodes	20,355	19,689	19,982
Total edges	4,797,639	2,610,183	2,098,516

**Table 2:** The pathway enrichment analysis by applying The Database for Annotation, Visualization and Integrated Discovery (DAVID) in core GWGEN of triple-negative breast cancer.

Pathway Analysis	Numbers	p-Value
Pathways in cancer	121	1.47 $\times 10^{-10}$
PI3K-Akt signaling pathway	102	5.30 $\times 10^{-8}$
MicroRNAs in cancer	81	9.38 $\times 10^{-6}$
MAPK signaling pathway	80	1.15 $\times 10^{-12}$
Transcriptional misregulation in cancer	77	2.24 $\times 10^{-11}$

**Table 3:** The pathway enrichment analysis by applying The Database for Annotation, Visualization and Integrated Discovery (DAVID) in core GWGEN of non-triple-negative breast cancer.

Pathway Analysis	Numbers	*p*-Value
Pathways in cancer	108	3.27 $\times 10^{-9}$
PI3K-Akt signaling pathway	104	1.60 $\times 10^{-8}$
MicroRNAs in cancer	85	3.02 $\times 10^{-6}$
Transcriptional misregulation in cancer	77	5.18 $\times 10^{-5}$
T cell receptor signaling pathway	53	3.80 $\times 10^{-4}$

**Table 4:** Potential multi-molecule drugs and their corresponding target genes for TNBC. ●: The targets of the potential multi-molecule drugs.

	Drugs	BRCA1	AKT1	FOXC1	MMP2	ETS1	STAT3
Targets							
Resveratrol		●		●		●	●
Sirolimus			●				
Prednisolone				●	●	●	●

**Table 5:** Potential multiple-molecular drugs and their corresponding target genes for non-TNBC. ●: The targets of the potential multi-molecule drugs.

	Drugs	BRCA1	AKT1	FOXC1	MMP2	NFE2L1
Targets						
Resveratrol		●		●		●
Sirolimus			●			
Carbamazepine				●	●	
Verapamil				●		●

## Data Availability

The TNBC and non-TNBC microarray data could be found in GSE41998, GSE32646, and GSE25066.

## Supplementary Materials

The following are available online at https://www.mdpi.com/1422-0067/22/6/3083/s1.
