# Supplementary material for: Systems Medicine Design for Triple-Negative Breast Cancer and Non-Triple-Negative Breast Cancer Based on Systems Identification and Carcinogenic Mechanisms
Source: Int J Mol Sci. 2021 Mar 17;22(6):3083. doi: 10.3390/ijms22063083 (PMC8002730; doi:10.3390/ijms22063083)
Supplement: Supplementary file 1 [file ijms-22-03083-s001.pdf]

Supplementary Materials

1.1 Systems modeling of miRNAs and lncRNAs

For lncRNAs regulatory model, the y-th lncRNA in the sample n is given as bellow:

$$l_y[n] = \sum_{u=1}^{U_y} \delta_{yu} t_u[n] + \sum_{\substack{k=1 \\ k \neq y}}^{K_y} \Gamma_{yk} l_k[n] - \sum_{v=1}^{V_y} \tau_{yv} m_v[n] l_y[n] + \psi_{\mathrm{y}} + \theta_{\mathrm{y}}[n], \quad (1)$$
$$\text{for } y = 1, \dots, Y,\ n = 1, \dots, N$$

where $l_y[n]$ represents the expression level of the y-th lncRNA; U_y indicates the total number of TFs binding to the y-th lncRNA; K_y represents the total number of lncRNAs binding to the y-th lncRNA; V_y denotes the total number of miRNAs inhibiting the y-th lncRNA; δ_{yu} denotes the transcription regulatory ability from the u-th TF to the y-th lncRNA; Γ_{yk} is the transcription regulatory ability from the k-th lncRNA to the y-th lncRNA; $\tau_{yv} \geq 0$ represents the post-transcription regulatory ability of the v-th miRNA inhibiting the y-th lncRNA; $t_u[n]$, $l_k[n]$, and $m_v[n]$ indicate the expression level of the u-th TF, the k-th lncRNA, and the v-th miRNA, respectively; Y is the total number of lncRNAs and N denotes the total number of data samples; ψ_{y} represents the basal level of the y-th lncRNA expression; $\theta_{\mathrm{y}}[n]$ is the stochastic noise of gene expression in the y-th lncRNA for the sample n caused by model uncertainty and data noise.

For miRNAs regulatory model, the z-th miRNA in the sample n could be formulated in the following form:

$$m_z[n]=\sum_{u=1}^{U_z}\upsilon_{zu}t_u[n]+\sum_{k=1}^{K_z}\sigma_{zk}l_k[n]-\sum_{\substack{v=1\\v\neq z}}^{V_z}\zeta_{zv}m_v[n]m_z[n]+\psi_z+\theta_z[n], \tag{2}$$

for $z=1,\ldots,Z,\ n=1,\ldots,N$

where $m_z[n]$ represents the expression level of the z-th miRNA; U_z indicates the total number of TFs binding to the z-th miRNA; K_z represents the total number of lncRNAs binding to the z-th miRNA; V_z denotes the total number of miRNAs inhibiting the z-th miRNA; υ_{zu} denotes the regulatory ability from the u-th TF to the z-th miRNA; σ_{zk} is the regulatory ability from the k-th lncRNA to the z-th miRNA; $\zeta_{zv}\geq 0$ represents the post-transcription regulatory ability of the v-th miRNA inhibitig the z-th miRNA; $t_u[n]$, $l_k[n]$, and $m_v[n]$ indicate the expression level of the u-th TF, the k-th lncRNA, and the v-th miRNA, respectively; Z is the total number of miRNAs and N is the total number of data samples; ψ_z represents the basal level of the z-th miRNA expression; θ_z[n] is the stochastic noise of gene expression in the z-th miRNA for the sample n including model uncertainty and data measurement noise.

1.2 Systems identification and model order selection for obtaining real GWGENs of TNBC and non-TNBC

To identify parameters in the miRNAs and lncRNAs regulatory model, we have to solve constraint least-square problems. Here, we have to turn miRNA regulation equation (1) into the linear regression form as below:

$$l_y[n]=\left[t_1[n]\ \cdots\ \mathrm{t}_{U_y}[n]\ l_1[n]\ \cdots\ l_{K_y}[n]\ m_1[n]\,l_y[n]\ \cdots\ m_{V_y}[n]\,l_y[n]\ \ 1\right]\times\begin{bmatrix}\delta_{y1}\\ \vdots\\ \delta_{yU_y}\\ \Gamma_{y1}\\ \vdots\\ \Gamma_{yK_y}\\ -\tau_{y1}\\ \vdots\\ -\tau_{yV_y}\\ \psi_y\end{bmatrix}+\theta_y\left[n\right] \quad (3)$$

$$=\Phi_{y,L}[n]\cdot\omega_{y,L}+\theta_y\left[n\right],\ \text{for } y=1,\ldots,Y.$$

where $\Phi_{\mathrm{y,L}}[\mathrm{n}]$ indicates the regression vector which can be gotten from the microarray data and $\omega_{\mathrm{x},G}$ represents the unknown parameter vector for the *y*-th lncRNA in GRN. The equation (3) of the *y*-th lncRNA could be augmented for *N* samples in the following form:

$$\begin{bmatrix}l_y[1]\\ l_y[2]\\ \vdots\\ l_y[\mathrm{N}]\end{bmatrix}=\begin{bmatrix}\Phi_{y,L}[1]\\ \Phi_{y,L}[2]\\ \vdots\\ \Phi_{y,L}[\mathrm{N}]\end{bmatrix}\cdot\omega_{y,L}+\begin{bmatrix}\theta_y\left[1\right]\\ \theta_y\left[2\right]\\ \vdots\\ \theta_y\left[N\right]\end{bmatrix},\ y=1,\ldots,Y,\ n=1,\ldots,N. \quad (4)$$

The equation (4) could be simply described as

$$\mathrm{L}_y=\Omega_{y,L}\cdot\omega_{y,L}+\vartheta_y \quad (5)$$

where

$$L_y = \begin{bmatrix} l_y[1] \\ l_y[2] \\ \vdots \\ l_y[\mathrm{N}] \end{bmatrix},\ \Omega_{y,L} = \begin{bmatrix} \Phi_{y,L}[1] \\ \Phi_{y,L}[2] \\ \vdots \\ \Phi_{y,L}[\mathrm{N}] \end{bmatrix},\ \vartheta_y = \begin{bmatrix} \theta_y[1] \\ \theta_y[2] \\ \vdots \\ \theta_y[N] \end{bmatrix} \tag{6}$$

Hence, by solving the constrained linear least-square problem in (7), the estimated regulatory parameters in the vector $\hat{\omega}_{\mathrm{y,L}}$ could be obtained. In the meanwhile, the miRNA repression parameters $-\tau_{\mathrm{y},v}$ are guaranteed to be non-positive, i.e., $-\tau_{\mathrm{y},v} \le 0$ for $v = 1,...,V_x$.

$$\hat{\omega}_{y,L} = \min_{\omega_{y,L}} \frac{1}{2} \left\| \Omega_{y,L} \cdot \omega_{y,L} - \mathrm{L}_y \right\|_2^2,$$
$$\text{subject to} \underbrace{\begin{bmatrix} 0 & \cdots & \cdots & 0 \\ \vdots & \ddots & & \vdots \\ \vdots & & \ddots & \vdots \\ 0 & \cdots & \cdots & 0 \end{bmatrix}}_{U_y} \left| \underbrace{\begin{matrix} 0 & \cdots & \cdots & 0 \\ \vdots & \ddots & & \vdots \\ \vdots & & \ddots & \vdots \\ 0 & \cdots & \cdots & 0 \end{matrix}}_{K_y} \right| \underbrace{\begin{matrix} 1 & 0 & \cdots & 0 \\ 0 & \ddots & \ddots & \vdots \\ \vdots & \ddots & \ddots & 0 \\ 0 & \cdots & 0 & 1 \end{matrix}}_{V_y} \left| \begin{matrix} 0 \\ \vdots \\ \vdots \\ 0 \end{matrix} \right] \omega_{y,L} \le \begin{bmatrix} 0 \\ \vdots \\ \vdots \\ 0 \end{bmatrix} \tag{7}$$

The linear regression form of the miRNA regulatory equation (2) in GRN could be rewritten in the following:

$$m_z[n]=\left[t_1[n]\ \cdots\ \mathrm{t}_{U_y}[n]\ l_1[n]\ \cdots\ l_{K_y}[n]\ m_1[n]\ m_z[n]\ \cdots\ m_{V_x}[n]\ m_z[n]\ \ 1\right]\times\begin{bmatrix}\upsilon_{z1}\\ \vdots\\ \upsilon_{zU_z}\\ \sigma_{z1}\\ \vdots\\ \sigma_{z\mathrm{K}_z}\\ -\zeta_{z1}\\ \vdots\\ -\zeta_{zV_z}\\ \psi_z\end{bmatrix}+\theta_z[n] \tag{8}$$

$$=\Phi_{z,M}[n]\cdot\omega_{z,M}+\theta_z[n],\ \text{for } z=1,\ldots,Z.$$

where $\Phi_{\mathrm{z,M}}[\mathrm{n}]$ indicates the regression vector which can be obtained from the microarray data and $\omega_{\mathrm{z,M}}$ represents the unknown parameter vector for the *z*-th miRNA in GRN. The equation (7) of the *z*-th miRNA could be augmented for *N* samples in the following form:

$$\begin{bmatrix}m_z[1]\\ m_z[2]\\ \vdots\\ m_z[\mathrm{N}]\end{bmatrix}=\begin{bmatrix}\Phi_{z,M}[1]\\ \Phi_{z,M}[2]\\ \vdots\\ \Phi_{z,M}[\mathrm{N}]\end{bmatrix}\cdot\omega_{z,M}+\begin{bmatrix}\theta_z[1]\\ \theta_z[2]\\ \vdots\\ \theta_z[N]\end{bmatrix},\ \text{for } z=1,...,Z,\ n=1,...,N. \tag{9}$$

The equation (8) could be simply described as

$$\mathrm{M}_z=\Omega_{z,M}\cdot\omega_{z,M}+\vartheta_z \tag{10}$$

where

$$M_z = \begin{bmatrix} m_z[1] \\ m_z[2] \\ \vdots \\ m_z[\mathrm{N}] \end{bmatrix},\ \Omega_{z,M} = \begin{bmatrix} \Phi_{z,M}[1] \\ \Phi_{z,M}[2] \\ \vdots \\ \Phi_{z,M}[\mathrm{N}] \end{bmatrix},\ \vartheta_z = \begin{bmatrix} \theta_z[1] \\ \theta_z[2] \\ \vdots \\ \theta_z[N] \end{bmatrix} \tag{11}$$

Therefore, by solving the constrained linear least-square problem in (12), estimated vector $\hat{\omega}_{\mathrm{z,M}}$ with the regulatory parameters could be obtained via MATLAB optimization toolbox. In the meanwhile, the miRNA repression parameters $-\zeta_{\mathrm{z},v}$ are guaranteed to be non-positive, i.e., $-\zeta_{\mathrm{z},v} \le 0$ for $v = 1,...,V_x$.

$$\hat{\omega}_{z,M} = \min_{\omega_{z,M}} \frac{1}{2} \left\| \Omega_{z,M} \cdot \omega_{z,M} - \mathrm{M}_z \right\|_2^2,$$
$$\text{subject to} \underbrace{\begin{bmatrix} 0 & \cdots & \cdots & 0 \\ \vdots & \ddots & & \vdots \\ \vdots & & \ddots & \vdots \\ 0 & \cdots & \cdots & 0 \end{bmatrix}}_{U_z} \underbrace{\begin{matrix} 0 & \cdots & \cdots & 0 \\ \vdots & \ddots & & \vdots \\ \vdots & & \ddots & \vdots \\ 0 & \cdots & \cdots & 0 \end{matrix}}_{K_z} \underbrace{\begin{matrix} 1 & 0 & \cdots & 0 \\ 0 & \ddots & \ddots & \vdots \\ \vdots & \ddots & \ddots & 0 \\ 0 & \cdots & 0 & 1 \end{matrix}}_{V_z} \begin{matrix} 0 \\ \vdots \\ \vdots \\ 0 \end{matrix} \; \omega_{z,M} \le \begin{bmatrix} 0 \\ \vdots \\ \vdots \\ 0 \end{bmatrix} \tag{12}$$

It is noted that miRNAs regulatory model and lncRNAs regulatory model exists false positives which might be caused by various experimental conditions. Therefore, we have to apply AIC to do model selection for pruning false positives. The AIC equation for the lncRNAs regulatory model is defined in the following:

$$AIC(U_y, K_y, V_y) = \log(\hat{\varepsilon}_{y,L}^2) + \frac{2(\Delta_{y,L})}{N},$$
$$\text{where } \hat{\varepsilon}_{y,L} = \sqrt{\frac{(L_y - (\Omega_{y,L} \cdot \hat{\omega}_{x,G}))^T (L_y - (\Omega_{y,L} \cdot \hat{\omega}_{y,L}))}{N}} \quad, \Delta_{y,L} = U_y + K_y + V_y + 1. \tag{13}$$

$\hat{\varepsilon}^2_{y,L}$ and $\Delta_{y,L}$ represent the estimated residual error and the number (order) of parameters for the y-th lncRNA, respectively; $\hat{\omega}_{y,L}$ is the estimated vector of the y-th lncRNA in (7). We would have the minimization of $AIC(U_y{}^*, K_y{}^*, V_y{}^*)$ when the real system order U_y^*, K_y^*, V_y^* are found. In other words, the insignificant interactions which are out of real system order U_y^*, K_y^*, V_y^* would be pruned away.

The AIC equation for the miRNAs regulatory model is defined in the following:

$$\begin{aligned} &\mathrm{AIC}(\mathrm{U_z},\mathrm{K_z},\mathrm{V_z})=\log(\hat{\varepsilon}^2_{\mathrm{z,M}})+\frac{2(\Delta_{\mathrm{z,M}})}{\mathrm{N}}, \\ &\text{where } \hat{\varepsilon}_{\mathrm{z,M}}=\sqrt{\frac{(\mathrm{M_z}\text{-}(\Omega_{\mathrm{z,M}}\times\hat{\omega}_{\mathrm{z,M}}))^{\mathrm{T}}(\mathrm{M_z}\text{-}(\Omega_{\mathrm{z,M}}\times\hat{\omega}_{\mathrm{z,M}}))}{\mathrm{N}}}\ ,\Delta_{\mathrm{z,M}}=\mathrm{U_z}+\mathrm{K_z}+\mathrm{V_z}+1. \end{aligned} \tag{14}$$

$\hat{\varepsilon}^2_{z,M}$ and $\Delta_{z,M}$ denote the estimated residual error and the number (order) of parameters for the z-th miRNA in (14), respectively; $\hat{\omega}_{z,M}$ is the estimated vector of the z-th miRNA in (12). The real system order U_z^*, K_z^*, V_z^* would make us have the minimization of $AIC(U_z{}^*, K_z{}^*, V_z{}^*)$.

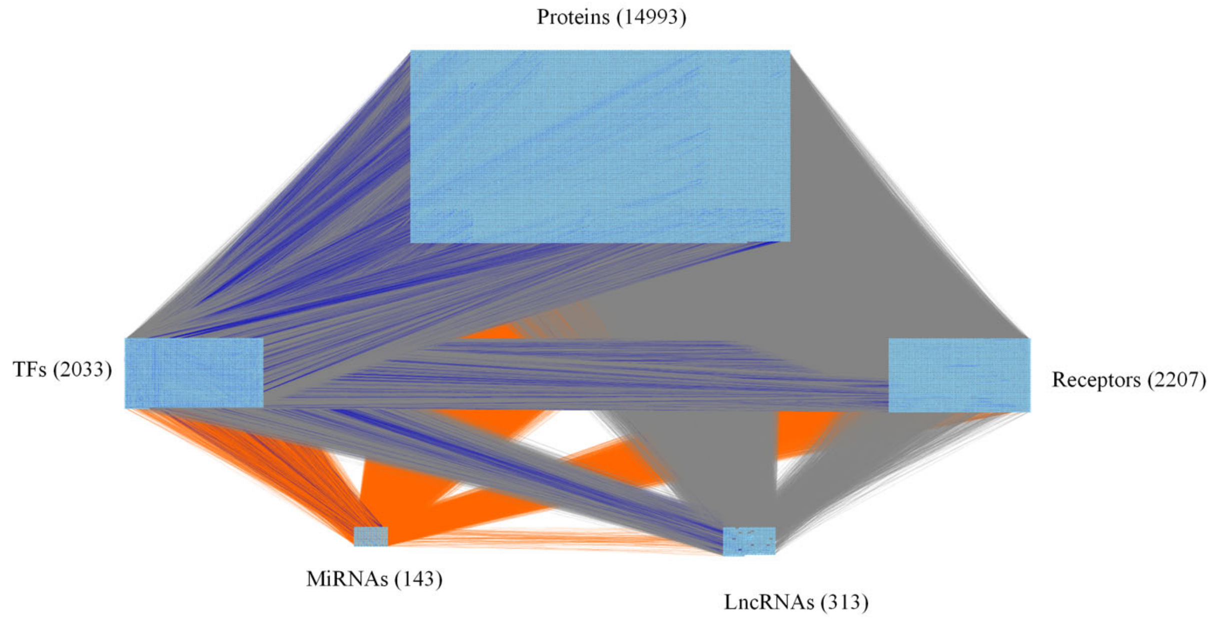

Figure S1. The real genome-wide genetic and epigenetic network (GWGEN) of TNBC.

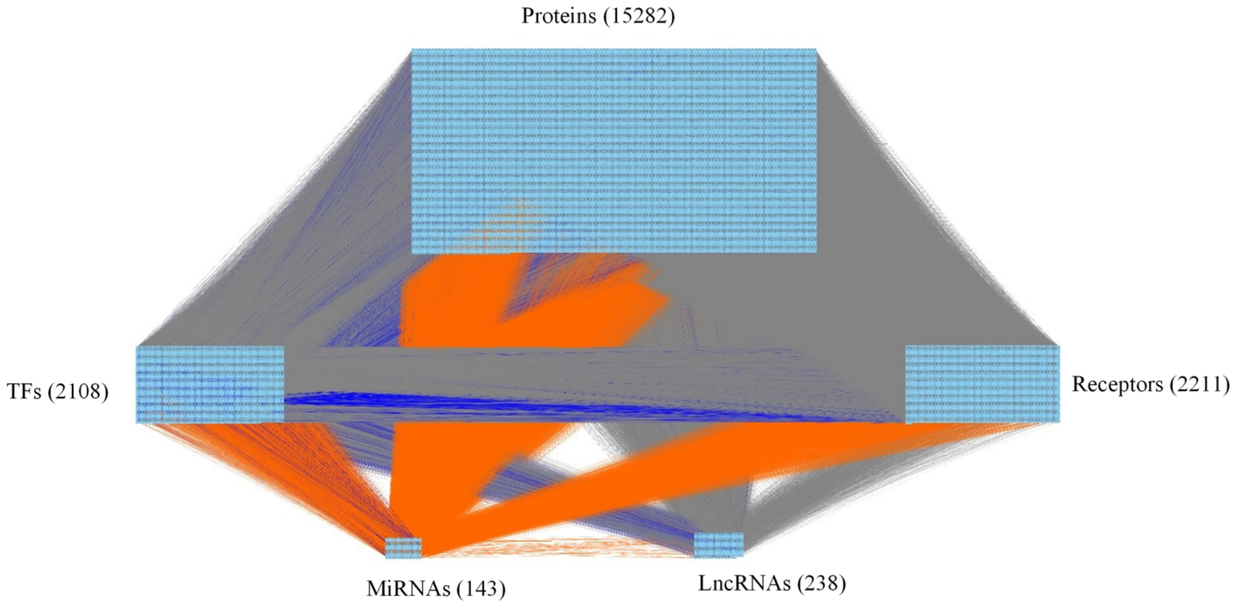

Figure S2. The real genome-wide genetic and epigenetic network (GWGEN) of non-TNBC.

Table S1. Candidate drugs filtered by regularability with LD50 for the selected essential drug targets.

BRCA1			AKT1		
Drug	Toxicity (LD50, mol/kg)	Regularability (CMap)	Drug	Toxicity (LD50, mol/kg)	Regularability (CMap)
Resveratrol	1.6791	-0.860	Danazol	1.3291	-0.060
Alsterpaullone	2.4368	-0.221	Norethisterone	1.8788	-0.146
Irinotecan	2.796	-0.257	Dacarbazine	1.9602	-0.074
Vinblastine	2.9111	-0.239	Probucol	1.9829	-0.921
Indometacin	4.0722	-0.405	Sirolimus	2.869	$-9.05*10^{-17}$

ETS1			FOXC1		
Drug	Toxicity (LD50, mol/kg)	Regularability (CMap)	Drug	Toxicity (LD50, mol/kg)	Regularability (CMap)
Resveratrol	1.6791	-0.622	Sulfasalazine	1.4383	-0.999
Prednisolone	1.8914	-0.670	Resveratrol	1.6791	-0.868
Carbamazepine	2.1131	-0.740	Prednisolone	1.8914	-0.564
Diclofenac	3.6447	-0.677	Carbamazepine	2.1131	-0.681
-	-	-	Verapamil	3.4137	-0.999

STAT3			MMP2		
Drug	Toxicity (LD50, mol/kg)	Regularability (CMap)	Drug	Toxicity (LD50, mol/kg)	Regularability (CMap)
Resveratrol	1.6791	-0.628	Prednisolone	1.8914	-0.885
Prednisolone	1.8914	-0.874	Tamoxifen	1.9882	-0.208
Carbamazepine	2.1131	-0.939	Carbamazepine	2.1131	-0.679
Novobiocin	2.2899	-0.985	Dexamethasone	2.1482	-0.576
-	-	-	Lomustine	3.5549	-0.357

NFE2L1		
Drug	Toxicity (LD50, mol/kg)	Regularability (CMap)
Resveratrol	1.6791	-0.814
Probucol	1.9829	-0.949
Carbamazepine	2.1131	-0.806
Sulindac	3.0989	-0.698
Verapamil	3.4137	-0.953
